# The Role of Neck Input in Producing Corrective Saccades in the Head Impulse Test

**DOI:** 10.3389/fneur.2022.881411

**Published:** 2022-05-17

**Authors:** Shinichi Iwasaki, Teru Kamogashira, Chisato Fujimoto, Kayoko Kabaya, Makoto Kinoshita, Tatsuya Yamasoba

**Affiliations:** ^1^Department of Otolaryngology & Head and Neck Surgery, Nagoya City University Graduate School of Medicine, Nagoya, Japan; ^2^Department of Otolaryngology and Head and Neck Surgery, Faculty of Medicine, The University of Tokyo, Tokyo, Japan

**Keywords:** vestibular, head impulse test, neck input, overt saccades, covert saccades

## Abstract

**Background:**

The head impulse test is a valuable clinical test that can help identify peripheral vestibular dysfunction by observing corrective saccades that return the eyes to the target of interest. Corrective saccades have been classified as covert if the onset occurs before the end of the head impulse and as overt if they occur afterwards. However, the mechanism that trigger these saccades remain unclear.

**Objective:**

The objective of this study was to examine the role of neck input in generating overt as well as covert saccades.

**Methods:**

Sixteen patients (9 males and 7 females: age 35-80 years, average 62.7 years old) who showed corrective saccades during the head impulse test were included. Twelve patients had unilateral vestibular dysfunction, and 4 patients had bilateral vestibular dysfunction. Patients underwent both the head impulse test (HIT) and the body impulse test (BIT) in a randomized order. While the head is rotated horizontally in HIT, the body is rotated horizontally in BIT. During BIT, the neck is fixed by a cervical collar (neck lock extrication collar) to reduce somatosensory input from the neck. The head movements and eye movements were recorded and analyzed by the video HIT recording system.

**Results:**

In all 16 patients, corrective saccades were observed in HIT as well as in BIT. While there were no significant differences in peak head velocities between HIT and BIT (*p* = 0.33, paired *t*-test), the VOR gain in BIT was significantly smaller than that in HIT (*p* = 0.011, paired *t*-test). The number of overt saccades per trial in BIT was significantly decreased compared to that in HIT (*p* < 0.001, paired *t*-test) whereas there were no significant differences in the number of covert saccades between the two tests. The proportion of overt saccades among all corrective saccades in BIT was significantly lower than the proportion in HIT (*p* < 0.001, paired *t*-test).

**Conclusions:**

Somatosensory input from the neck contributes to the generation of overt saccades and reinforces the vestibulo-ocular reflex complementing the retinal slip during high frequency head movements.

## Introduction

The head impulse test (HIT) is a valuable clinical test to identify peripheral vestibular dysfunction (1, 2). When clinicians turn the head briskly in the plane of the semicircular canals, healthy subjects can stabilize their gaze with the angular vestibulo-ocular reflex (VOR) that compensates for the head rotation with an equal eye rotation in the direction opposite to the head. In patients with vestibular dysfunction, the eyes move with the head, requiring corrective saccades to return the gaze to the earth-fixed target. Detecting these corrective saccades is the clinical sign of VOR hypofunction in clinical HIT (2).

Objective eye movement measurement during HIT using the scleral search coil technique as well as high-speed video imaging has revealed that corrective saccades occur not only after the end of the eye response defined as the zero crossing of eye velocity but also during the head rotation (3, 4). The latter is called a covert saccade, since it is difficult to perceive them without recording equipment. The former is called an overt saccade since it is easily observed by the clinician without recording.

The mechanisms of how corrective saccades are generated are still unclear. Previous studies have suggested that they are triggered by retinal slip (5), visual input (6), residual vestibular function (7), or internal models created in the central nervous system (8). However, the role of somatosensory input from cervical movement in the generation of corrective saccades is still unclear.

Previous studies have shown that while the contribution of the somatosensory input from cervical movement to VOR is minimal in normal conditions, it potentiates after bilateral vestibular loss (9, 10). After unilateral labyrinthectomy, somatosensory input can be used to change activity in the vestibular afferents in frogs (11), but not in primates (12, 13).

To investigate the role of cervical input in generating corrective saccades in HIT, we performed both HIT and the body impulse test (BIT), in which the examiner quickly rotates the patient's body with their neck fixed using a cervical collar, in patients with peripheral vestibular dysfunction. We compared the differences in the generation of corrective saccades between HIT and BIT and revealed that the sensory input from the neck mainly contributes to the generation of overt saccades.

## Methods

### Experimental Procedure

All procedures were in accordance with the Helsinki declaration and were approved by the University of Tokyo Human Ethics Committee (No. 2487). All patients gave written informed consent.

### Patients

Sixteen patients (9 males and 7 females; age 35–80 years, mean age 62.7 ± 14.6 years) were recruited from the Balance Disorder Clinic, Department of Otolaryngology at the University of Tokyo Hospital. All the patients received a detailed history taking and neuro-otological tests including the head impulse test, caloric test (4°C ice water). The methods of these vestibular function tests have been described previously (14). The inclusion criteria were (1) unilateral or bilateral vestibular hypofunction shown with the caloric test (canal paresis calculated using Jongkee's formula >20%, or maximum slow phase eye velocity < 10°/s bilaterally) (14), (2) corrective saccades present with decreased VOR gain (<0.8) on at least on one side in the lateral canal using vHIT (15), and (3) free from any disease of the central nervous system. The clinical characteristics of the patients are listed in Table 1. The vestibular schwannomas in patients #10 and #11 were located in the internal auditory meatus without any compression of the brainstem. The tumors sizes were 6.2 and 4.7 mm, respectively.

**Table 1:** Clinical characteristics of the 16 patients with vestibular dysfunction.

**Patient No**.	**Age**	**Sex**	**Affected side**	**Etiologies**	**Duration from onset**	**vHIT gain**		**Caloric (deg/s)**	
						**Deficit**	**Normal**	**Deficit**	**Normal**
1	36	F	R	Vestibular neuritis	3 months	0.27	0.46	0	25
2	51	M	R	Vestibular neuritis	38 days	0.22	0.83	0	11
3	53	M	R	Vestibular neuritis	10 days	0.37	0.98	4.8	29
4	54	F	L	Vestibular neuritis	1 year	0.32	0.81	3.9	15
5	75	F	R	Vestibular neuritis	40 days	0.53	1.06	3.4	23
6	75	F	L	Vestibular neuritis	8 months	0.57	0.84	3.3	50
7	80	M	L	Vestibular neuritis	2.6 years	0.52	1.18	4.3	21
8	77	F	R	Meniere's disease	4 years	0.73	0.83	4	18
9	78	F	L	Meniere's disease	2 months	0.67	1.11	22	41
10	35	M	L	Vestibular schwannoma	>10 years	0.27	0.77	0	24.8
11	64	F	L	Vestibular schwannoma	>10 years	0.76	1.1	3.6	15
12	67	M	L	Labyrinthitis	1.5 years	0.55	0.97	0	14
						**R**	**L**	**R**	**L**
13	51	M	Bilateral	Aminoglycoside	2.2 years	0.53	0.51	1.3	0.5
14	61	M	Bilateral	Aminoglycoside	10 months	0.72	0.69	0	2.5
15	70	M	Bilateral	Unknown	10 months	0.23	0.25	0	0
16	76	M	Bilateral	A3243G	Unknown	0.5	0.62	1.4	2.2

*R, right; L, left; vHIT, video head impulse test; A3243G, mitochondrial A3243G mutation*.

### Head Impulse Test (HIT)

**Figure 1:**
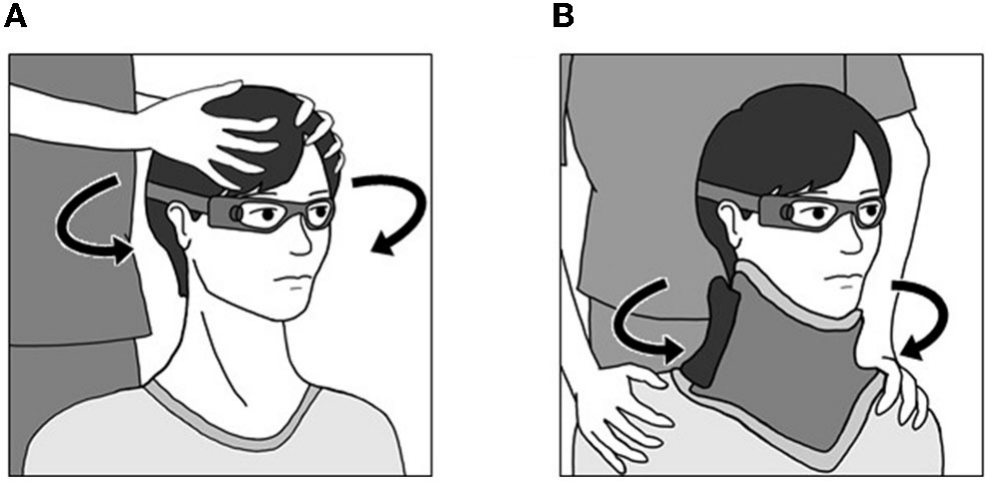
Methods for the head impulse test and body impulse test. **(A)** Head Impulse Test. The examiner stood behind the subject and held the subject's head. Horizontal head impulses were manually applied to each side with unpredictable timing and direction by the examiner. **(B)** Body Impulse Test. Subjects were seated on a rotating stool, and wore a cervical collar to minimize neck movements. The examiner held both shoulders. Impulsive horizontal body rotation was manually applied to each side with unpredictable timing and direction by the examiner.

Subjects were seated on a chair in a light room, and were instructed to visualize an eye level target on the wall at a distance of 1 m. The examiner stood behind the subject and held the subject's head. Horizontal head impulses were manually applied to each side with unpredictable timing and direction by the examiner (Figure 1A). The amplitude of the head rotation was 15-20°, and the head velocity of the impulse was 110-220°/s. At least 20 accepted head impulses were collected for each horizontal semicircular canal.

### Body Impulse Test (BIT)

Subjects were seated on a rotating stool, the seat of which could rotate freely in the horizontal plane, and were instructed to visualize an eye level target on the wall at a distance of 1 m in a light room. They wore a cervical collar (Ossur Miami-J Neck Brace; Ossur Japan, Tokyo) to stabilize the head. To minimize neck movement, particular care was taken to secure the neck brace tightly around the neck. The examiner stood behind the subject and held both shoulders. Impulsive horizontal body rotations were manually applied to each side with unpredictable timing and direction by the examiner (Figure 1B). The amplitude of the rotation was 15-20°. The head velocity of the impulse was 110-220°/s. At least 20 accepted head impulses were collected for each horizontal semicircular canal.

HIT and BIT were performed in a randomized order.

### Data Collection and Analysis

Eye velocity and head velocity were recorded for each head rotation using an Otometrics Impulse device (Otometrics, Denmark). Subjects wore a lightweight goggle frame with a built-in camera to record eye movements from the right eye and an accelerometer to record head movement at a sampling frequency of 250 Hz. The values of VOR gain were calculated as the ratio of mean eye velocity over mean head velocity during a 40-ms window centered at peak head acceleration (3).

Corrective saccades were analyzed off-line with custom SciLab 6.1.1 software (SciLab, ESI Group). Saccades were identified by their peak velocity. We only analyzed corrective saccades that brought the eye toward the target. Compensatory saccades that occurred during the head movement were classified as “covert.” Saccades appearing after the head movement had finished were classified as “overt” (4). The beginning of a head impulse was defined as the first time point when the head velocity exceeded 2% of the peak prior to peak head velocity. The end of a head impulse was defined as the first time when head velocity had a zero crossing after the peak velocity. The amplitude of the saccade was measured at the peak. Covert saccade amplitude was adjusted such that velocity of the VOR was removed (16). The latency of the saccade was the difference between the beginning of the head impulse and the time of the peak velocity of the saccade.

### Statistical Analysis

Data are expressed as mean ± SD. Statistical analysis of the data was done using SPSS software version 21 (IBM Corp., Armonk, NY, USA). The peak head velocity, VOR gain, and the number, latency and amplitude of corrective saccades between HIT and BIT were compared using a paired *t*-test. The correlation between the decrease in the proportion of overt saccades and various parameters (duration from the onset, HIT gain, differences in gain between HIT and BIT, difference in head velocities between HIT and BIT, and age) were examined using a Pearson's correlation coefficient analysis. A difference was considered significant at *p* < 0.05.

## Results

**Figure 2:**
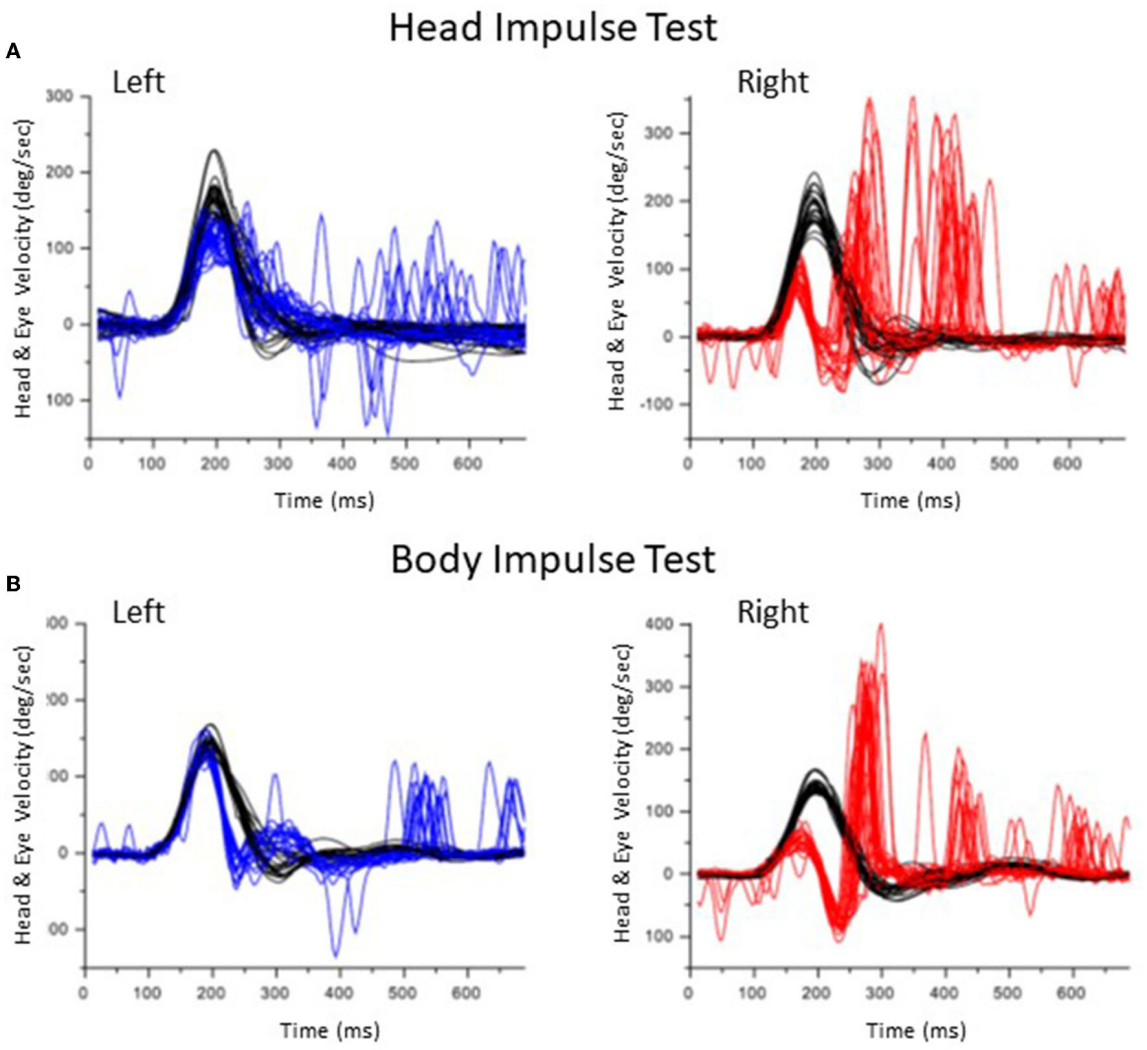
Results of the head impulse test and body impulse test in a patient with a right vestibular neuritis (Patient #2). **(A)** Head Impulse test. Head velocity is black. Eye velocity is blue in the left lateral rotation and red in the right lateral rotation. In the right lateral rotation, both covert and overt saccades were observed. **(B)** Body Impulse Test. In the right lateral rotation, overt saccades decreased.

Figure 2 shows the results of HIT and BIT in a representative patient with vestibular neuritis on the right side (Patient #2 in Table 1). While both overt and covert saccades were observed in HIT to the right side, almost all saccades were covert in BIT to the same side.

We performed HIT as well as BIT in 12 patients with unilateral vestibulopathy and 4 patients with bilateral vestibulopathy. Disruptive artifacts such as a large bounce or multiple peaks (17) were not observed either in HIT or BIT. Since there were no significant differences in the decrease in the proportion of overt saccades in BIT compared to HIT between patients with unilateral vestibular dysfunction and bilateral vestibular dysfunction (*p* = 0.079, unpaired *t*-test), we combined the data of the patients with unilateral and bilateral vestibular dysfunction.

Table 2 shows the summarized results of HIT and BIT in 20 affected sides in total. While there were no significant differences in peak head velocities between HIT and BIT (*p* = 0.33, paired *t*-test), the VOR gain in BIT was significantly smaller than that in HIT (*p* = 0.011, paired *t*-test). The number of corrective saccades in BIT was significantly smaller than that in HIT (*p* = 0.0011, paired *t*-test). There were no significant differences in the number of covert saccades per trial between HIT and BIT (*p* = 0.81, paired *t*-test). However, the number of overt saccades in BIT was significantly decreased compared to that in HIT (*p* < 0.001, paired *t*-test). The proportion of covert saccades among all corrective saccades in HIT was 54.3 ± 11.4% whereas in BIT it was significantly greater at 74.2 ± 14.9% (*p* < 0.001, paired *t*-test). The latency of all corrective saccades in BIT was significantly smaller than that in HIT (*p* = 0.0065, paired *t*-test).

**Table 2:** Results of head impulse test and body impulse test.

	**Head impulse test (*n* = 20 ears)**	**Body impulse test (*n* = 20 ears)**	***p*-value**
Peak head velocity (deg/s)	166.4 ± 46.5	155.7 ± 36.9	0.33
Gain	0.50 ± 0.19	0.42 ± 0.17	0.011^*^
Number of all corrective saccades (per trial)	2.14 ± 0.53	1.59 ± 0.49	0.0011^**^
Number of covert saccades (per trial)	1.15 ± 0.35	1.13 ± 0.23	0.81
Number of overt saccades (per trial)	0.99 ± 0.38	0.46 ± 0.37	<0.001^***^
Proportion of covert saccades (%)	54.3 ± 11.4	74.2 ± 14.9	<0.001^***^
Proportion of overt saccades (%)	45.7 ± 11.4	25.8 ± 14.9	<0.001^***^
Latency of all corrective saccades (ms)	244.7 ± 25.0	214.1 ± 50.9	0.0065^**^
Velocity of all corrective saccades (deg/s)	127.1 ± 27.4	122.3 ± 33.0	0.49
Velocity of covert saccades (deg/s)	123.0 ± 19.4	125.1 ± 35.9	0.76
Velocity of overt saccades (deg/s)	130.5 ± 37.4	117.0 ± 29.3	0.081

* *p < 0.05*,

** *p < 0.01*,

*** *p < 0.001*.

Since we included patients with a variety of diseases, we also analyzed the results of HIT and BIT limited to patients with vestibular neuritis (Patients #1-#7; Supplementary Table 1). While there were no significant differences in peak head velocities between HIT and BIT (*p* = 0.34, paired *t*-test), the VOR gain in BIT was significantly smaller than that in HIT (*p* = 0.028, paired *t*-test). The proportion of covert saccades among all corrective saccades in BIT (67.3 ± 11.4%) significantly increased compared to than that in HIT (57.3 ± 14.1%; *p* = 0.038, paired *t*-test).

To investigate the factors that have an association with the decrease in the proportion of overt saccades in BIT in comparison with HIT, we examined its correlation with several factors (Supplementary Figure 1). There were no significant correlations between the decrease in the rate of overt saccades in BIT and the duration from the onset of the disease (*r* = 0.039, *p* = 0.89, Pearson's correlation coefficient analysis), the gain in HIT (*r* = 0.13, *p* = 0.58, Pearson's correlation coefficient analysis), differences in gain in between HIT and BIT (*r* = −0.057, *p* = 0.81, Pearson's correlation coefficient analysis) or the difference in head velocity between HIT and BIT (*r* = 0.31, *p* = 0.19, Pearson's correlation coefficient analysis). Only patient age had a weak correlation with the decrease in the rate of overt saccades in BIT (*r* = −0.453, *p* = 0.045, Pearson's correlation coefficient analysis).

## Discussion

In the present study, we performed HIT as well as BIT in patients with peripheral vestibular dysfunction, and showed that the proportion of overt saccades significantly decreased in BIT compared to HIT, suggesting that input from the neck contributes to the production of overt saccades in HIT.

During each head impulse, corrective saccades are induced to bring the eyes back on target, and they have been accepted as a clinical sign of vestibular dysfunction. Corrective saccades have been suggested to have a role in facilitating patient recovery (9). Visual processing is suppressed during saccades, so that neither the motion of the eye nor the blur of the retinal image during inadequate VOR is perceived. This saccadic suppression can reduce oscillopsia and improve visual performance during high-velocity head movements (9, 10).

The mechanism of how corrective saccades are generated is still unclear. Retinal slip during head rotation has been suggested as the main trigger of corrective saccades (5, 6). However, the presence of corrective saccades in darkness indicates that there are triggers other than the retinal slip during head movement (6). The remaining vestibular function and the somatosensory input from the neck have been proposed as possible triggers of corrective saccades (18). Another possible explanation might be generation through internal models in the central nervous system (8). This hypothesis is consistent with previous findings that corrective saccades are triggered earlier when the head turns are active (19, 20), and when they are predictable (21).

We have addressed the question of whether the somatosensory input from the neck contributes to the generation of corrective saccades during high-velocity head rotations. When the head is rotated on the body, neck joints and muscle proprioceptors are also activated, generating the slow phase eye movement through the cervico-ocular reflex (COR) pathway. While the contribution of the COR to gaze stabilization is very small under normal conditions, it has been shown that the COR potentiates after bilateral vestibular loss (22, 23). Since this potentiated COR mainly compensates for head velocity at lower frequencies, the slow phase eye movement generated by the COR may contribute to improving the slow compensatory eye responses to passive low-acceleration head turns but not to generating the slow eye movements to passive head turns with naturally high accelerations. However, it is possible that the potentiated COR enhances oculomotor responses to low frequency stimuli and serves to trigger covert saccadic responses for high-acceleration head rotations. In fact, it has been reported that a patient with absent labyrinthine function was able to make both the slow and quick phases of nystagmus during rotation of the body with the head stationary in darkness (24). In the present study, BIT showed slightly but significantly reduced gain in comparison with HIT, suggesting that the somatosensory input from the neck can contribute to generating compensatory eye movements to the high-frequency head movement.

During head rotation, gaze shifts are achieved by eye-head saccades to bring the image of an object to the fovea (25, 26). The eye-head saccades are composed of eye and head components which show distinct properties. The head component lags the eye component by ~40 ms although this latency can be affected by the shape of the driving neural signal (27). The waveform of the head component is different from that of the eye component and its velocity increases with the amplitude of the head movement. The generator of the head component is considered to be distinct from that of the eye component, and includes the superior colliculus in the brainstem (25). It is possible that the head component of eye-head saccades may contribute to the generation of overt saccades in HIT since the proportion of overt saccades was significantly reduced in BIT compared to HIT.

There are several limitations in the present study. First, the sample size was relatively small, which might make it difficult to analyze the factors that have an association with the decrease in the proportion of overt saccades in BIT in comparison with HIT. Second, we included patients with wide range of duration from the onset of disease. It has been reported that the gain of the COR continues to increase after making vestibular lesions (23). However, there were no significant correlations between the duration from the onset of the disease and the decrease in the proportion of overt saccades in BIT compared to HIT. Third, the body rotation in BIT was not measured. There is a possibility that there are differences in velocities of body movement and that of head movement in BIT. Additionally, it is possible that the cervical collar used while performing BIT may not completely eliminate the somatosensory input from the neck. We carefully secured the collar to stabilize the head on the body to reduce the movement of the neck during the body rotation. It is possible that the pressure of the collar to the neck skin and muscle might affect the COR and the generation of corrective saccades.

In conclusion, we have performed BIT and HIT in patients with unilateral and bilateral vestibular dysfunction, and shown that stabilization of the neck resulted in a reduction of overt saccades and a slight decrease in the VOR gain. These results suggest that the somatosensory input from the neck contributes to the generation of overt saccades and reinforces the VOR, complementing the retinal slip trigger during high frequency head movements.

## Data Availability Statement

The raw data supporting the conclusions of this article will be made available by the authors, without undue reservation.

## Ethics Statement

The studies involving human participants were reviewed and approved by the University of Tokyo Human Ethics Committee. The patients/participants provided their written informed consent to participate in this study.

## Author Contributions

SI, TK, CF, KK, and TY conceived and designed the experiments, wrote, and revised the manuscript. SI, TK, CF, and MK performed and analyzed the experiments. All authors contributed to the article and approved the submitted version.

## Funding

This work was supported by the Ministry of Education, Culture, Sports, Science and Technology (18K09370, 21H03088) and the Japan Agency for Medical Research and Development (AMED).

## Conflict of Interest

The authors declare that the research was conducted in the absence of any commercial or financial relationships that could be construed as a potential conflict of interest.

## Publisher's Note

All claims expressed in this article are solely those of the authors and do not necessarily represent those of their affiliated organizations, or those of the publisher, the editors and the reviewers. Any product that may be evaluated in this article, or claim that may be made by its manufacturer, is not guaranteed or endorsed by the publisher.

## Supplementary Material

Supplementary Figure 1 Correlation of the decrease in the proportion of overt saccades in BIT in comparison with HIT with various parameters. **(A)** Correlation between the decrease in the proportion of overt saccades in BIT in comparison with HIT and the duration from the onset of disease. **(B)** Correlation between the decrease in the proportion of overt saccades in BIT in comparison with HIT and VOR gain in HIT. **(C)** Correlation between the decrease in the proportion of overt saccades in BIT in comparison with HIT and the differences in gain between HIT and BIT. **(D)** Correlation between the decrease in the proportion of overt saccades in BIT in comparison with HIT and the differences in head velocities between HIT and BIT. **(E)** Correlation between the decrease in the proportion of overt saccades in BIT in comparison with HIT and the patient's age.

Supplementary Table 1 Results of head impulse test and body impulse test in patients with vestibular neuritis.

## References

[1] Halmagyi GM Chen L MacDougall HG Weber KP McGarvie LA Curthoys IS. The video head impulse test. Front Neurol. (2017) 8:258. 10.3389/fneur.2017.00258 28649224 PMC5465266

[2] Halmagyi GM Curthoys IS. A clinical sign of canal paresis. Arch Neurol. (1988) 45:737–9. 10.1001/archneur.1988.00520310043015 3390028

[3] MacDougall HG Weber KP McGarvie LA Halmagyi GM Curthoys IS. The video head impulse test: diagnostic accuracy in peripheral vestibulopathy. Neurology. (2009) 73:1134–41. 10.1212/WNL.0b013e3181bacf85 19805730 PMC2890997

[4] Weber KP Aw ST Todd MJ McGarvie LA Curthoys IS Halmagyi GM. Head impulse test in unilateral vestibular loss: vestibulo-ocular reflex and catch-up saccades. Neurology. (2008) 70:454–63. 10.1212/01.wnl.0000299117.48935.2e 18250290

[5] Scherer M Schubert MC. High-velocity angular vestibulo-ocular reflex adaptation to position error signals. J Neurol Phys Ther. (2010) 34:82–6. 10.1097/NPT.0b013e3181dde7bc 20588093 PMC2954113

[6] Van Nechel C Bostan A Duquesne U Hautefort C Toupet M. Visual input is the main trigger and parametric determinant for catch-up saccades during video head impulse test in bilateral vestibular loss. Front Neurol. (2018) 9:1138. 10.3389/fneur.2018.01138 30662427 PMC6328459

[7] Tian J Crane BT Demer JL. Vestibular catch-up saccades in labyrinthine deficiency. Exp Brain Res. (2000) 131:448–57. 10.1007/s002219900320 10803413

[8] Colagiorgio P Versino M Colnaghi S Quaglieri S Manfrin M Zamaro E. New insights into vestibular-saccade interaction based on covert corrective saccades in patients with unilateral vestibular deficits. J Neurophysiol. (2017) 117:2324–38. 10.1152/jn.00864.2016 28404827 PMC5491708

[9] Leigh RJ Zee DS. The Saccadic System. The Neurology of Eye Movement. 5 ed. Oxford University Press (2015). p. 169-288.

[10] Matin E. Saccadic suppression: a review and an analysis. Psychol Bull. (1974) 81:899–917. 10.1037/h0037368 4612577

[11] Precht W Llinás R Clarke M. Physiological responses of frog vestibular fibers to horizontal angular rotation. Exp Brain Res. (1971) 13:378–407. 10.1007/bf00234338 5123644

[12] Cullen KE Minor LB Beraneck M Sadeghi SG. Neural substrates underlying vestibular compensation: contribution of peripheral versus central processing. J Vestib Res. (2009) 19:171–82. 10.3233/ves-2009-0357 20495234 PMC3319765

[13] Sadeghi SG Minor LB Cullen KE. Dynamics of the horizontal vestibuloocular reflex after unilateral labyrinthectomy: response to high frequency, high acceleration, and high velocity rotations. Exp Brain Res. (2006) 175:471–84. 10.1007/s00221-006-0567-7 16957885

[14] Fujimoto C Suzuki S Kinoshita M Egami N Sugasawa K Iwasaki S. Clinical features of otolith organ-specific vestibular dysfunction. Clin Neurophysiol. (2018) 129:238–45. 10.1016/j.clinph.2017.11.006 29207275

[15] McGarvie LA MacDougall HG Halmagyi GM Burgess AM Weber KP Curthoys IS. The video head impulse test (vHIT) of semicircular canal function - age-dependent normative values of vor gain in healthy subjects. Front Neurol. (2015) 6:154. 10.3389/fneur.2015.00154 26217301 PMC4495346

[16] Anson ER Bigelow RT Carey JP Xue QL Studenski S Schubert MC. Aging increases compensatory saccade amplitude in the video head impulse test. Front Neurol. (2016) 7:113. 10.3389/fneur.2016.00113 27486430 PMC4947583

[17] Mantokoudis G Saber Tehrani AS Kattah JC Eibenberger K Guede CI Zee DS. Quantifying the vestibulo-ocular reflex with video-oculography: nature and frequency of artifacts. Audiol Neuro otol. (2015) 20:39–50. 10.1159/000362780 25501133

[18] Macdougall HG Curthoys IS. Plasticity during vestibular compensation: the role of Saccades. Front Neurol. (2012) 3:21. 10.3389/fneur.2012.00021 22403569 PMC3289127

[19] Black RA Halmagyi GM Thurtell MJ Todd MJ Curthoys IS. The active head-impulse test in unilateral peripheral vestibulopathy. Arch Neurol. (2005) 62:290–3. 10.1001/archneur.62.2.290 15710858

[20] Peng GC Minor LB Zee DS. Gaze position corrective eye movements in normal subjects and in patients with vestibular deficits. Ann. N Y Acad. Sci. (2005) 1039:337–48. 10.1196/annals.1325.032 15826987

[21] Mantokoudis G Agrawal Y Newman-Toker DE Xie L Saber Tehrani AS Wong A. Compensatory saccades benefit from prediction during head impulse testing in early recovery from vestibular deafferentation. Eur Arch Otorhinolaryngol. (2016) 273:1379–85. 10.1007/s00405-015-3685-7 26088345

[22] Bronstein AM. Plastic changes in the human cervicoocular reflex. Ann. N Y Acad Sci. (1992) 656:708–15. 10.1111/j.1749-6632.1992.tb25248.x 1599175

[23] Yakushin SB Kolesnikova OV Cohen B Ogorodnikov DA Suzuki J Della Santina CC. Complementary gain modifications of the cervico-ocular (COR) and angular vestibulo-ocular (aVOR) reflexes after canal plugging. Exp Brain Res. (2011) 210:549–60. 10.1007/s00221-011-2558-6 21286691 PMC3134235

[24] Kasai T Zee DS. Eye-head coordination in labyrinthine-defective human beings. Brain Res. (1978) 144:123–41. 10.1016/0006-8993(78)90439-0 638756

[25] Guitton D. Control of eye-head coordination during orienting gaze shifts. Trends Neurosci. (1992) 15:174–9. 10.1016/0166-2236(92)90169-9 1377424

[26] Proudlock FA Gottlob I. Physiology and pathology of eye-head coordination. Prog Retin Eye Res. (2007) 26:486–515. 10.1016/j.preteyeres.2007.03.004 17548226

[27] Zangemeister WH Stark L. Gaze latency: variable interactions of head and eye latency. Exp Neurol. (1982) 75:389–406. 10.1016/0014-4886(82)90169-8 7106221

