# Supplementary figures and images for: The Role of Neck Input in Producing Corrective Saccades in the Head Impulse Test
Source: Front Neurol. 2022 May 17;13:881411. doi: 10.3389/fneur.2022.881411 (PMC9152213; doi:10.3389/fneur.2022.881411)

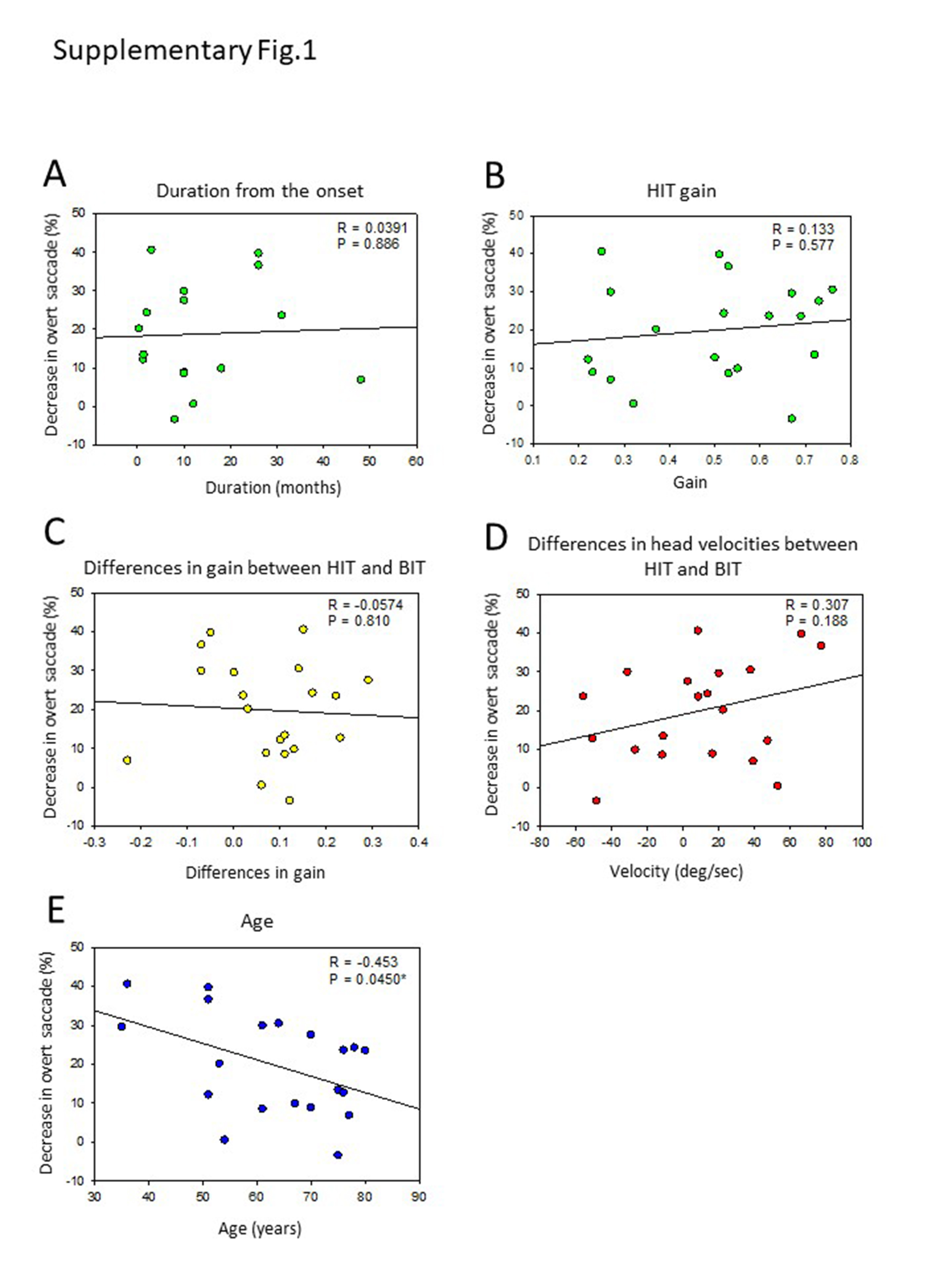

Supplement: Supplementary Figure 1 — Correlation of the decrease in the proportion of overt saccades in BIT in comparison with HIT with various parameters. (A) Correlation between the decrease in the proportion of overt saccades in BIT in comparison with HIT and the duration from the onset of disease. (B) Correlation between the decrease in the proportion of overt saccades in BIT in comparison with HIT and VOR gain in HIT. (C) Correlation between the decrease in the proportion of overt saccades in BIT in comparison with HIT and the differences in gain between HIT and BIT. (D) Correlation between the decrease in the proportion of overt saccades in BIT in comparison with HIT and the differences in head velocities between HIT and BIT. (E) Correlation between the decrease in the proportion of overt saccades in BIT in comparison with HIT and the patient's age. [file Image_1.JPEG]
