# Supplementary material for: The Role of Neck Input in Producing Corrective Saccades in the Head Impulse Test
Source: Front Neurol. 2022 May 17;13:881411. doi: 10.3389/fneur.2022.881411 (PMC9152213; doi:10.3389/fneur.2022.881411)
Supplement: Supplementary Table 1 — Results of head impulse test and body impulse test in patients with vestibular neuritis. [file Table_1.DOCX]

| **Supplementary Table 1. Results of head impulse test and body impulse test in patients with vestibular neuritis** | | | |
| --- | --- | --- | --- |
|  | Head Impulse Test (n = 7 ears) | Body Impulse Test (n = 7 ears) | p-value |
| Peak head velocity (deg/sec) | 188.9 ± 30.8 | 172.6 ± 20.0 | 0.34 |
| Gain | 0.41 ± 0.16 | 0.36 ± 0.15 | 0.028* |
|  |  |  |  |
| Number of all corrective saccades (per trial) | 2.42 ± 0.77 | 1.91 ± 0.52 | 0.17 |
| Number of covert saccades (per trial) | 1.36 ± 0.47 | 1.24 ± 0.20 | 0.52 |
| Number of overt saccades (per trial) | 1.06 ± 0.54 | 0.67 ± 0.42 | 0.080 |
| Proportion of covert saccades (%) | 57.3 ± 14.1 | 67.3 ± 12.9 | 0.038* |
| Proportion of overt saccades (%) | 42.7 ± 14.1 | 32.7 ± 12.9 | 0.038* |
|  |  |  |  |
| Latency of all corrective saccades (ms) | 245.8 ± 31.6 | 227.7 ± 42.1 | 0.24 |
| Latency of covert saccades (ms) | 159.6 ± 15.1 | 155.4 ± 13.5 | 0.34 |
| Latency of overt saccades (ms) | 363.7 ± 38.9 | 348.6 ± 55.5 | 0.49 |
|  |  |  |  |
| Velocity of all corrective saccades (deg/s) | 166.1 ± 28.1 | 132.3 ± 31.6 | 0.70 |
| Velocity of covert saccades (deg/s) | 173.0 ± 26.9 | 136.3 ± 55.7 | 0.50 |
| Velocity of overt saccades (deg/s) | 148.6 ± 46.0 | 124.0 ± 32.9 | 0.15 |
| * p < 0.05, | | | |
